# Model-guided fieldwork: practical guidelines for multidisciplinary research on wildlife ecological and epidemiological dynamics

**DOI:** 10.1111/j.1461-0248.2012.01836.x

**Published:** 2012-07-19

**Authors:** Olivier Restif, David T S Hayman, Juliet R C Pulliam, Raina K Plowright, Dylan B George, Angela D Luis, Andrew A Cunningham, Richard A Bowen, Anthony R Fooks, Thomas J O'Shea, James L N Wood, Colleen T Webb

**Affiliations:** 1 Disease Dynamics Unit, Department of Veterinary Medicine, University of CambridgeMadingley Road, Cambridge, CB3 0ES, UK; 2 Department of Biology, Colorado State UniversityFort Collins, CO, 80523, USA; 3 Institute of Zoology, Zoological Society of LondonLondon, NW1 4RY, UK; 4 Animal Health and Veterinary Laboratories AgencyWeybridge, Surrey, KT15 3NB, UK; 5 Fogarty International Center, National Institutes of HealthBethesda, MD, 20892, USA; 6 Department of Biology, University of FloridaGainesville, FL, 32611, USA; 7 Emerging Pathogens Institute, University of FloridaGainesville, FL, 32610, USA; 8 Center for Infectious Disease Dynamics, The Pennsylvania State UniversityUniversity Park, PA, 16802, USA; 9 Department of DefenseFrederick, MD, 21702, USA; 10 Department of Biomedical Sciences, Colorado State UniversityFort Collins, CO, 80523, USA; 11 The National Centre for Zoonosis Research, University of LiverpoolLeahurst, Neston, South Wirral, CH64 7TE, UK

**Keywords:** Field ecology, infectious diseases, mathematical models, statistical models, study design, wildlife epidemiology

## Abstract

Infectious disease ecology has recently raised its public profile beyond the scientific community due to the major threats that wildlife infections pose to biological conservation, animal welfare, human health and food security. As we start unravelling the full extent of emerging infectious diseases, there is an urgent need to facilitate multidisciplinary research in this area. Even though research in ecology has always had a strong theoretical component, cultural and technical hurdles often hamper direct collaboration between theoreticians and empiricists. Building upon our collective experience of multidisciplinary research and teaching in this area, we propose practical guidelines to help with effective integration among mathematical modelling, fieldwork and laboratory work. Modelling tools can be used at all steps of a field-based research programme, from the formulation of working hypotheses to field study design and data analysis. We illustrate our model-guided fieldwork framework with two case studies we have been conducting on wildlife infectious diseases: plague transmission in prairie dogs and lyssavirus dynamics in American and African bats. These demonstrate that mechanistic models, if properly integrated in research programmes, can provide a framework for holistic approaches to complex biological systems.

## Introduction

Concluding a report on the controversy surrounding Ross and Waite's pioneering mathematical models for malaria transmission, the British Medical Journal pointed out, in 1911, ‘the paradox that quantitative work based on false postulates may, by stimulating biologists and field workers to a closer scrutiny of the facts, sometimes lead more certainly to the discovery of the truth, than non-quantitative investigations resting on impeccable foundations’. A hundred years later, and despite the ubiquity of mathematical models in all fields of life sciences, the statement retains remarkable pertinence. By describing mathematically the unobserved mechanisms hypothesised to be causing biological phenomena (e.g. the boom-and-bust nature of epidemics or the cyclical fluctuations of animal populations) in a Newtonian fashion, Ross and other early modellers finally brought life sciences on a par with physical sciences. Mechanistic models have come to form the backbone of modern teaching and research in ecology. The mainstream use of mathematical models in ecology, sometimes referred to as strategic modelling (Gurney & Nisbet 1998), aims to formulate simple descriptions of universal drivers of population dynamics. Over the last century, this approach has provided useful insight into general principles of ecology and has helped ecologists generate testable hypotheses. However, it is not always clear to scientists working on a particular system what added value such generic models can bring. This stems from the conceptual, technical and, at times, cultural difficulties faced when trying to match models with empirical information, as experienced by anyone working at the interface of theoretical and empirical research.

In principle, mathematical models can be embedded in broader frameworks for scientific investigation based on hypothesis generation and experimental falsification or validation. It is almost 50 years since Platt (1964) laid out the principles of strong inference and advocated an iterative process of formulating multiple alternative hypotheses, generating testable predictions, gathering experimental evidence and then revisiting the hypotheses in view of the evidence. Along the same lines, mechanistic models should evolve with the experimental evidence generated through the scientific process. Various textbooks, such as Hilborn & Mangel's (1997) Ecological Detective, have greatly contributed to promoting model integration in population ecology. This iterative process has been put into practice in ecology in various contexts, notably experimental planktonic prey–predator systems, where the systematic use of mathematical models has helped ascertain and quantify the respective roles of diverse factors, including resource availability (Fussmann *et al*. 2000) and genetic composition (Becks *et al*. 2010), in the generation of complex trophic dynamics. Extending such approaches to natural populations poses many challenges, but there are examples of iterative implementation of observations, theory and experiments in field settings. Many of these have aimed to determine the drivers of population cycles, following a tradition set by Volterra (1926), and have encompassed a broad range of ecological factors and interactions: from the iconic Canadian lynx-hare predator–prey system (Krebs *et al*. 2001) to the Soay sheep population of St Kilda island (Coulson *et al*. 2001), and from the British red grouse and its parasites (Hudson *et al*. 1998) to the California red scale (a pest of citrus trees) and its parasitoid (Murdoch *et al*. 2006). However, the common thread to all these examples is that only after long time series (several years if not decades) of data had been collected did theoretical questions appear, leading ultimately to a cycle of mathematical models and experimental validation. Ecologists have generally failed to harness the power of mechanistic models for study design and data integration during early phases of field studies, which can limit the power of data analysis and inference at later stages.

What can we learn from retrospective studies to improve the flow of exchange between empirical and theoretical methods at the onset of a new programme of field-based research? This question is particularly topical in infectious disease ecology. Indeed, beyond the academic motivation of integrating pathogens into ecological frameworks (Lafferty *et al*. 2008), wildlife infections have recently received a surge of attention in broader scientific and political communities because of the various threats they pose at the global level (Daszak *et al*. 2000): to the conservation of the species affected (Blaustein & Kiesecker 2002; Haydon *et al*. 2006; Frick *et al*. 2010); to ecosystem stability (Rizzo & Garbelotto 2003); to the viability and trade of livestock infected from wildlife reservoirs (Siembieda *et al*. 2011) and to public health in human populations affected by zoonotic diseases (Kuiken *et al*. 2005). From a scientific point of view, infectious diseases in wildlife are particularly challenging ecological systems because their dynamics are determined by processes operating at multiple scales (Table 1), and because of practical difficulties with data collection in populations that are often difficult to observe or sample. Understanding these key processes requires input from many disciplines, using multiple methodologies and analysing diverse datasets to triangulate the causal drivers of disease dynamics (Plowright *et al*. 2008).

**Table 1:** Multiple scales at which the dynamics of wildlife infectious diseases can be modelled

Level	Example	References
Within host	Effect of maternal antibodies on demography	Kallio *et al*. (2010)
Within groups of hosts	Social network governing infectious contacts between animals	Drewe (2010)
Between groups	Metapopulation dynamics	Haydon *et al*. (2006)
Across landscapes	Spatiotemporal waves of infection guided by natural barriers	Russell *et al*. (2005)
Between host species	Environmental reservoirs	Haydon *et al*. (2002)
	Pathogen-mediated competition	Tompkins *et al*. (2003)
Between parasite species	Interactions within parasite communities	Telfer *et al*. (2010)
From wildlife to humans	Risk factors for zoonotic emergence	Jones *et al*. (2008)

In this study, we emphasise the benefits of integrating mathematical and statistical models with empirical and experimental approaches at all stages of a research project, with the first iteration preceding data collection. This not only improves hypothesis generation and study design but also increases the quantity and quality of information gained from empirical studies. To this aim, we present a practical framework called model-guided fieldwork (MGF), which demands a rational dialogue between researchers from multiple disciplines through a series of iterative steps, ultimately leading to improved causal inference and predictive power. While the vast majority of ecologists are aware of the usefulness of mathematical models, a lack of interdisciplinary expertise within research teams all too often prevents models from being used to their full potential. We aim to provide guidelines for ‘good practice’ in multidisciplinary ecological research, largely inspired by our own experience in wildlife disease ecology.

**Figure 1:**
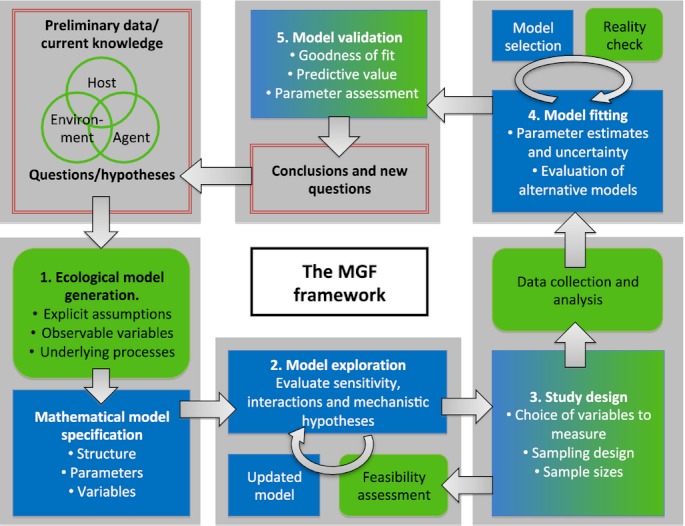
Schematic diagram of our model-guided fieldwork framework, emphasising feedback loops between empiricists and modellers. Numbers refer to the five key steps described in the text; however, it is possible to initiate the collaborative cycle at any stage. Dark grey boxes represent tasks led by modellers, light grey rounded boxes tasks led by biologists and a gradient indicates shared responsibility (online version in colour: blue for modellers’ tasks, green for biologists’ tasks).

In the MGF framework, biologists and modellers collaborate at all stages of the study, from initial model formulation and field study design, to data collection and analysis. The MGF approach recognises that there is often uncertainty in system structure and drivers, and utilises *a priori* mechanistic models to ensure field efforts can address this uncertainty. A key strength of the MGF approach is the planned iterative refinement of fieldwork, laboratory experiments and modelling throughout the project, ensuring empirical studies are more focused and models are data driven and appropriate to the specific system. Compared to after-the-fact modelling approaches, MGF helps to focus field studies on the most important structures and drivers of dynamics. Furthermore, the necessary continuous dialogue between collaborators throughout the project lifespan fosters a multidisciplinary, multidirectional flow of information (Fig. 1). It is important to underline that we envisage the framework shown in Fig. 1 as a strategic master plan that may unfold over several years, and which would be broken down into a number of smaller studies. However, it would be misleading to consider the large multidirectional framework as the mere juxtaposition of separate unidirectional studies. In the MGF programme, modellers and biologists are involved in all steps, creating feedback loops that are missing from too many studies.

Below we describe the MGF framework in detail, emphasising the practical contributions that modellers can make to the conception, design, implementation and analysis of field studies. As illustrated in Fig. 1, the five key stages presented here should not be seen as a linear process, but as steps on a cycle of interactions that can be initiated from any point and reiterated multiple times. We then present two case studies illustrating different components of the MGF framework. Finally, the discussion summarises the scientific benefits of the approach and casts light on the inner workings and the challenges faced when breaking barriers between traditional disciplines. The level of involvement required from all parties goes against the high level of specialisation prevalent in science; we provide some suggestions about planning and organisation in a multidisciplinary context, with insight from social sciences.

## Description of the MGF Framework

### Ecological model generation: from the conceptual to the mathematical

Mechanistic models are a formalisation of the hypothesised processes that drive the observed dynamics of a biological system. Some of these processes may be measured in real time in the field (e.g. births), others may be measured in controlled conditions (e.g. duration of infectious period), while others may not be observable (e.g. transmission of infection from individual to individual). The first step of MGF is to integrate the evidence-based, qualitative and quantitative descriptions of these processes into a formal mathematical model that attempts to describe the dynamics of observed variables—in disease ecology, typically numbers of individuals in different categories (e.g. age, sex, infectious or immunological status). Particularly when limited information is available about the nature of a process, one should consider multiple hypotheses in a strong inference approach (Platt 1964): instead of trying to falsify a single hypothesis, it is often more informative to formulate a comprehensive set of biologically plausible, alternative hypotheses and assess their relative merits to explain available data. For example, the relative importance of multiple routes of transmission (Webb *et al*. 2006; Rohani *et al*. 2009) or multiple drivers of epidemic cycles (Wearing & Rohani 2006) can be assessed using mechanistic modelling approaches once data have been collected. Although more heuristic methods for hypothesis generation can be used, MGF forces researchers to be extremely specific in detailing their questions of interest and underlying assumptions. This specificity helps to ensure that the data collected will be appropriate for the analyses planned later (Fig. 1).

Once a conceptual mechanistic model of the system has been proposed and formalised in diagrams and verbal description, theoreticians translate it into a mechanistic mathematical model, checking the appropriateness of every assumption with the interdisciplinary team. Where existing modelling frameworks do not appear suitable, novel, tailor-made model structures can be designed, or a combination of models may be used for different parts of the system. Designing model structures (i.e. equations describing the temporal or spatiotemporal changes in biological variables) will highlight essential parameters and appropriate methods of estimation: either direct measurement or indirect inference by fitting models to the data. Identification of the parameters in itself guides the experimental and field study design (Fig. 1).

Modellers must ensure that the parameters are correctly interpreted. For quantities that can be measured in the field, it is important to check that the mathematical parameters have the same dimension as the measurements. Conversely, when the model includes parameters that cannot be measured directly, the team should try to find practical ways in which the estimates of those parameters, once fitted, can be validated indirectly. For example, in a model for epidemics, the transmission rate in itself is typically of little practical use, but it can be combined with other parameters to form the basic reproductive rate of the epidemic, which has an intuitive interpretation and practical implications. Because the correct interpretation of a parameter depends on the way it is included mathematically in the equations, it is also important to discuss the choice of alternative functions describing key processes in the ecological system considered. Partially specified models (Wood 2001) can also be considered when there is uncertainty in the choice of mathematical functions.

In some cases, the choice of model structure may not be straightforward. For example, most models for the population dynamics of infectious diseases assume that individuals will go through a small number of discrete states during the process of infection, typically: susceptible (uninfected), latent (infected but not infectious), infectious and recovered (often with lifelong protective immunity). Even if the studied infection follows this general progression, individuals may not be unambiguously assigned to a single status (McClintock *et al*. 2010); for example, it is often impossible to know from a measured antibody titre whether the animal is currently infected or whether it is immune against future infection. Complementary experimental data are often needed to provide this kind of information, and further model refinement may be required to incorporate this biological richness (Charleston *et al*. 2011). The most relevant level of aggregation of individuals into discrete categories must be discussed carefully, taking into account the accuracy of the measurements available, the complexity of the model and the corresponding statistical methods. If not considered properly, these issues can lead to an ill-posed question, i.e. a situation where the results are of no practical use, as reviewed by Loehle (2011).

### Model exploration

Once a draft model is constructed, its dynamics must be explored over a wide range of parameter values and alternative assumptions using mathematical analysis and numerical simulations (e.g. Plowright *et al*. 2011). Patterns that the model can generate, and qualitative features expected in the data (Duke-Sylvester *et al*. 2010) can be identified, such as the existence and stability of equilibrium states, steady or waning oscillations (Hampson *et al*. 2007) or extinction of either the host or the infectious agent (Lloyd-Smith *et al*. 2005). As described in the next section, this initial analysis will inform the design of the field study (e.g. how many observations or samples are required to detect hypothesised changes in disease dynamics?), and can highlight flaws in the model (e.g. if it fails to replicate known dynamics, such as oscillations, or predicts an unrealistic prevalence of infection). This ‘feasibility assessment’ stage (Fig. 1) helps the scientific team identify aspects of the system that are poorly understood and need further empirical study or updates to the model structure.

Sensitivity analysis is another essential process that helps focus data collection effort on the most important parameters, by determining how changes in parameters affect model output (Blower & Dowlatabadi 1994; Cariboni *et al*. 2007). Highly sensitive parameters may require more measurement effort because the model output is more dependent on these parameters. From a mathematical viewpoint, sensitivity analysis should explore most of the parameter space. The biological range of some parameters can be quite wide, especially when considering a variety of environmental conditions. In many cases, however, the most relevant biological information results from a more local sensitivity analysis in the parameter space, which can be loosely determined from previous knowledge. Local sensitivity analysis is useful because there is often interdependence in parameter values and sensitivities, and this informed process can help to reduce data collection effort by focusing on the most relevant region of the parameter space. Determining interactions between parameters can also help pre-empt issues with identifiability that may appear at a later stage, by suggesting simplifications in the model. A typical example would be a pair of parameters governing reproduction and mortality in perfectly symmetric ways, which could result in population dynamics affected by the ratio (or the difference) of the two parameters rather than their absolute values: the pair of parameters can then be replaced with a single aggregate parameter.

### Study design

The design of ecological studies should aim to maximise the information that can be obtained from the data within the practical constraints imposed by the system. Although sample size calculations have become a standard practice in life sciences, mechanistic models are rarely used at this stage of empirical research. Once *a priori* models have been developed, as outlined in the previous section, they can help suggest how field data should be collected to optimise integration with other data sources. For example, De Jong & Bouma (2001) described a practical experimental framework for the measurement of vaccine-induced herd immunity in animal populations, based on a generic mathematical model for disease transmission.

Using mechanistic models ahead of field studies can improve the reliability of data collection. Craft *et al*. (2009) built a network model based on data describing the social interactions among Serengeti lions, and used it to run simulations of disease outbreaks. They quantified the impact of several properties of the empirically derived network on the outcome of the simulations, and highlighted potential biases caused by the way data had been collected. Although this analysis was conducted on a *post hoc* basis, such advice can help with the design of future field studies.

Furthermore, modelling exercises can suggest additional measurements that had been overlooked. Rohani *et al*. (2009) demonstrated theoretically that environmental transmission could play a more significant role than previously acknowledged during outbreaks of avian influenza, which should encourage measurements for the presence of influenza viruses in the environment. In another example, Plowright *et al*. (2011) developed a metapopulation model to simulate the dynamics of Hendra virus within fruit bat populations, thus providing a mechanistic explanation of increasing spillover from fruit bats into domestic horses in Australia. The inclusion of waning maternal immunity in the model improved the temporal match of simulated outbreaks to the observed ones. Although the presence of maternally derived antibody (MDA) has been reported, measurement of waning immunity itself in wild animals may not be feasible; instead experimental studies on captive bats could be carried out to test the hypothesis that MDA is protective and to derive empirical estimates of the rate of MDA decline.

### Model fitting

A crucial step of MGF comes when the model has been designed and the data have been collected: matching the two together with the help of statistical modelling. The first objective is usually to estimate the value of model parameters that were not known at the time of model construction. In the case where alternative hypotheses have been incorporated into different models, an additional objective is to assess which hypothesis provides the ‘best fit’ of the model to the data. What is meant by ‘best fit’ is an essential question that needs to be addressed by all the parties involved, biologists, modellers and statisticians, even before the data have been collected. Although a number of methods for curve fitting, such as smoothing, least squares or non-linear forecasting, have been traditionally employed for ecological time series (Kendall *et al*. 1999), they tend to consider the sources of error and variability as black boxes. In contrast, likelihood-based models can incorporate specific error-generating mechanisms (e.g. demographic stochasticity, sampling methods, imperfect assays), and therefore generate more reliable predictions (Clark & Bjørnstad 2004). Combined with information criteria (such as Akaike's Information Criterion), they also allow multiple model comparison and weighting (Burnham & Anderson 2001). Likelihood-based methods allow the computation of confidence intervals on parameter estimates, providing evidence to compare the relative importance of the mechanisms considered.

The last two decades have seen considerable advancement in the statistical methods available to fit dynamic models to empirical data, especially in the field of infectious diseases (Becker & Britton 1999). An increasingly popular approach is to use a Bayesian framework, which offers several advantages. First, all model parameters follow probability distributions rather than being treated as fixed quantities. This enables measures of parameter uncertainty to be generated. Second, the Bayesian framework allows prior information (e.g. parameter ranges obtained from available data sources, such as published papers) to be incorporated into the model structure. This is somewhat controversial as poor choices of priors can unduly influence the final estimates. However, where reliable information is available, a careful use of priors can help to ensure that the parameter estimates obtained are realistic. Indeed, certain combinations of parameter values that fall outside of meaningful biological ranges might happen to produce a perfect fit to the data by virtue of the mathematical properties of the model, but the use of prior information can help maintain parameters within biologically appropriate ranges. Third, in the Bayesian framework, any missing information in the data (e.g. due to incomplete observations or long time intervals between repeated measurements) can be treated as extra parameters in the model, and estimated as part of the model fitting process (O'Neill & Roberts 1999; Clark & Bjørnstad 2004). Recent methods have also been developed to deal with missing information within a frequentist framework (Ionides *et al*. 2006). Other new techniques, such as approximate Bayesian computation, provide a natural framework to estimate parameters in stochastic ecological models (Hartig *et al*. 2011), which are particularly relevant for infectious disease dynamics.

Whether using Bayesian or frequentist statistical models, a constant dialogue must be maintained between all parties involved. Indeed, the fitting process must be informed by the biologists to ensure the data are correctly interpreted and meaningfully analysed. It is not uncommon that data collection differs in various degrees from the initial plans because of logistical issues or unexpected field conditions. This may require a reassessment of the fitting procedures to account for missing data. It also is essential to submit every output from data analysis to a reality check by the field biologists: for example, unrealistic parameter estimates might reveal flaws in models, guiding the selection of alternative models or the revision of unsuitable assumptions. As a result, it is not unusual for the process of fitting models to data to take several months to complete. The numerical algorithms involved are often very complex, with risk of human error, and can take several days to run, even on modern computers.

### Model validation

The first step of validation is the assessment of the ‘goodness of fit’ of the model(s). Even though model fitting procedures aim to minimise the difference between observations and model prediction and to select the best-supported model, substantial discrepancies may remain. Statistical tests can be used to assess whether the remaining differences (or residuals) between the fitted model and the actual data may be attributed to random noise. However, such tests must be interpreted with caution: statistical support does not guarantee that the model assumptions are correct; conversely, a statistically significant discrepancy should not necessarily lead to a rejection of the mechanistic model as a whole. The value of any model lies in its ability to improve our understanding of specific processes, which does not necessarily require a perfect match to all the mechanisms of the real system. Therefore, a subjective assessment of model dynamics, informed by biological knowledge, remains important—this is another ‘reality check’ at the core of the MGF process (Fig. 1). Predictions from a fitted model should always be discussed critically in the context of both the model structure and the data collected.

The second step of validation confronts predictions of the model with an independent set of data, i.e. data not used in the fitting procedure. For example, if the same variables have been measured in two different locations that differ in known characteristics, once the model has been fitted to the data from one site, it can be used to predict the observations in the other location by modifying certain parameter values accordingly. Alternatively, where several variables have been measured in a single study, it is possible to fit the model to a subset of variables and then use the model to predict the dynamics of the remaining variables. If successful, this step is strong evidence that some fundamentally important aspects of the system have been captured by the mechanistic model. This allows the researchers to draw conclusions about the causes of observed patterns. Thus, once a valid model is produced, it will help assess the original set of hypotheses. If the model fails this step of validation, it is worth reconsidering alternative models that may have been proposed in the early stages and discarded on the basis of the original fitting procedure. However, as in the previous step of validation, some discrepancies should be expected when confronting model predictions to independent data: again, this should not trigger an automatic rejection of the model, but rather encourage a critical assessment of its assumptions through discussion between modellers and empirical biologists. In particular, the magnitude of the discrepancies that can be accepted should be informed by the biology of the system, by potential uncertainties introduced by data collection procedures, and by the type of model-generated predictions sought. This step of validation can result in modifications of the model as well as additional experiments; these iterative improvements are at the core of the MGF philosophy.

Lastly, a third component of model validation is an assessment of the relative importance of the model parameters. There are two relatively common approaches for this. The first one uses an information criterion framework that penalises the explanatory power of models by their complexity (number of parameters), as described in step 4 above for model selection. Once the best model has been identified, it may still be possible to simplify it further, e.g. by setting the values of some parameters to zero and assessing the effect on the information criterion. The second approach is to use sensitivity analysis on a single validated model to determine the relative importance of different processes incorporated in that single model. In this approach, parameters are associated with particular processes of interest (e.g. transmission pathways). Parameters that strongly affect the behaviour of models (i.e. which have high sensitivity) are associated with relatively important processes.

By submitting fitted models to detailed scrutiny, the scientific team should aim to identify any remaining discrepancies and issues, leading to further iterations of model improvement and data collection. Components of the models that failed the validation tests can be more informative than those that passed, which echoes the 1911 quote cited in the opening of this study. Indeed, a key aspect of MGF is that the model is not an end point. As in a strong inference perspective (Platt 1964), the inference drawn from the model should generate new questions and hypotheses that can be investigated through another cycle of MGF (Fig. 1).

## Case studies

**Figure 2:**
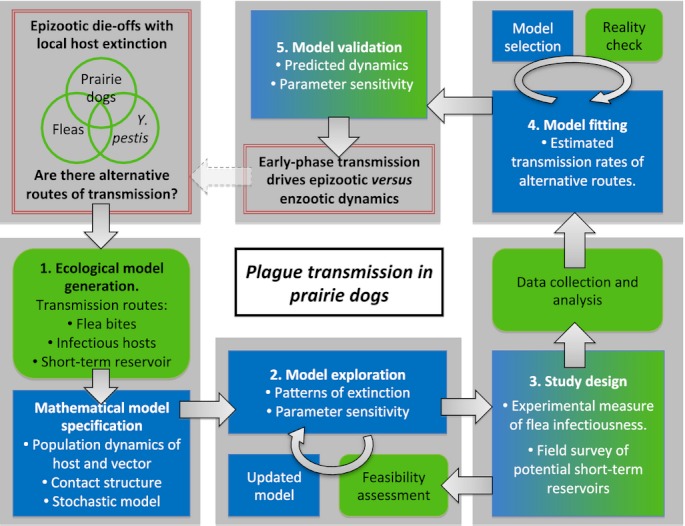
Application of MGF to plague (*Yersinia pestis*) in prairie dogs (*Cynomys ludovicianus*). The aim was to assess the relative importance of different routes of transmission of *Y. pestis* in causing observed extinctions of prairie dog towns.

**Figure 3:**
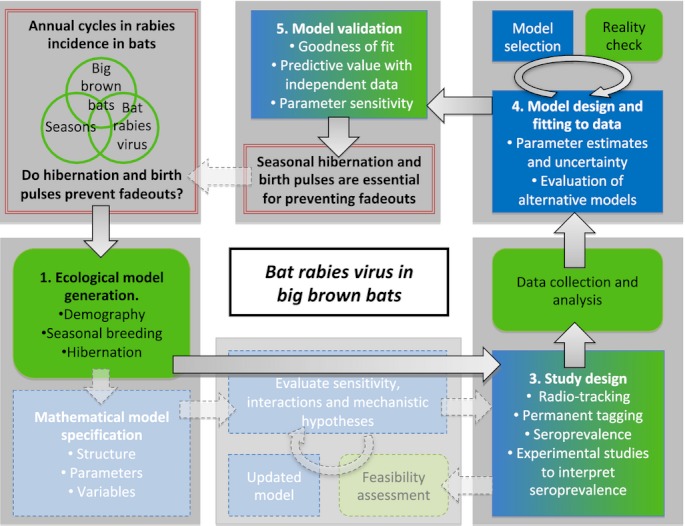
Application of MGF to bat rabies virus in big brown bats (*Eptesicus fuscus*). This study aimed at assessing the role of seasonal hibernation and birth pulses in the persistence of rabies virus in bat populations. Here, most data were collected before a formal mathematical model was developed.

**Figure 4:**
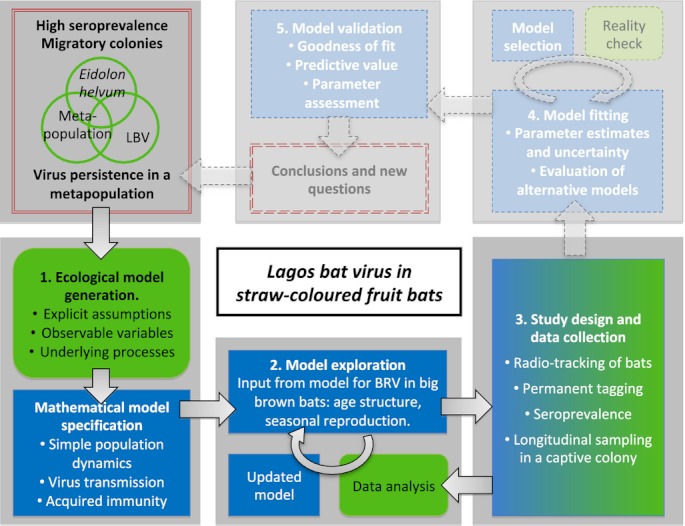
Application of MGF to Lagos bat virus (LBV) in straw-coloured fruit bats (*Eidolon helvum*). This ongoing research programme is investigating the interactions between bat life history (age structure, migrations, seasonal birth pulses) and the circulation of LBV. The modelling framework is being developed in conjunction with data collection and with input from the project on rabies virus in big brown bats.

The MGF framework represents a flexible and modular set of guidelines for ambitious, long-term research programmes in ecology. In most cases, such programmes will progress through a series of specific research objectives corresponding to subsets of the whole framework. Appreciating from the onset how mathematical models can contribute to those different steps is not an obvious task, but one that requires patience and commitment from all parties involved. As mentioned in the introduction, the MGF framework is not meant as a rigid, one-size-fits-all set of rules, but as a guideline for multidisciplinary integration. To demonstrate how MGF research can be implemented from different premises, we present detailed accounts of this process using two case studies from our own research. Whereas the first case study (plague transmission in prairie dogs, Fig. 2) followed steps 1–5 quite naturally, the second one (lyssaviruses in bats, Figs 3 and 4) combines two initially independent projects which started at different points along the cycle until it was realised they could complement each other within an MGF approach.

### Plague transmission in prairie dogs

Black-tailed prairie dogs (*Cynomys ludovicianus*) are extremely susceptible to plague and exhibit epizootic die-offs resulting in the apparent extinction of prairie dog towns. Researchers wished to determine the mechanisms underlying these spectacular die-offs to better understand how they occur and might be managed. According to the dominant paradigm in the literature, *Yersinia pestis*, the aetiological agent of plague, forms a biofilm that blocks the proventriculus of fleas feeding on mammals; transmission occurs when infectious bacteria from the blockage are regurgitated. Models that assumed blocked flea transmission as the only process predicted flea loads that were inconsistent with those observed in the field (Lorange *et al*. 2005; Webb *et al*. 2006). From these results and other observations, field researchers questioned the relevance of the dominant assumption to the prairie dog system, especially because the paradigm was based on transmission in a peridomestic system that differs in several important features from plague outbreaks in wildlife populations (Gage & Kosoy 2005).

Plague has a rich and long scientific literature including many older studies with valuable information from detailed laboratory and field observations for different species of rodent hosts and flea vectors. Under closer scrutiny by empirical researchers and modellers, the literature revealed an array of proposed transmission mechanisms that could broadly be classified into three types: blocked flea transmission, pneumonic transmission and transmission from a short-term reservoir. This led to the development of a mechanistic model that incorporated those three alternative transmission pathways (Webb *et al*. 2006). Most model parameters were taken from literature on prairie dogs or closely related species, although field data were used to estimate three remaining parameters using multiple methods including fitting procedures to the proposed model and estimation separate from the model. A stochastic version of the model was used to predict the probability of extinction of prairie dogs and fleas and the time to extinction. These predictions were validated using an independent 20-year data set of observed outbreaks. The model achieved a reasonable match to the observed data, and sensitivity analysis revealed that transmission from a short-term reservoir was the only route consistent with the observed data. This led to specific recommendations for data collection, initiating a new cycle of MGF (Fig. 2). In particular, multiple hypotheses were consistent with the short-term reservoir scenario and constraints on the infectious period of the short-term reservoir predicted by the model, including early-phase transmission before blockage occurred, transmission from carcasses and transmission from alternative infected hosts.

Based on the model, a series of experiments established that early-phase transmission was feasible in a laboratory setting (Eisen *et al*. 2006; Wilder *et al*. 2008), and measured the decay of infectiousness from carcasses. In parallel, data were also collected on infection rates in alternative hosts (Stapp & Salkeld 2009). This field and laboratory work corresponds to Phase 3 in MGF (Fig. 2). Finally, the new data were incorporated into more specific models that accounted for the newly proposed transmission mechanisms and which were then validated (Salkeld *et al*. 2010; Buhnerkempe *et al*. 2011), corresponding to Steps 4 and 5 in MGF (Fig. 2). The two different models that were developed need to be reconciled, but overall it appears likely that early-phase transmission drives the initial spread of epizootics with secondary roles for other transmission routes once host limitation occurs (Buhnerkempe *et al*. 2011).

### Seasonal dynamics of lyssaviruses in bats

The role of bats as reservoirs of zoonotic viral infections is increasingly recognised (Calisher *et al*. 2006). Rabies virus and related lyssaviruses are important pathogens of bat origin (Badrane & Tordo 2001); however, mechanisms of persistence of lyssaviruses in populations of bats and the drivers thereof have not been well described. We have recently led two projects investigating the effects of two different seasonal behaviours on bat lyssavirus infection dynamics: hibernation in a temperate bat species and migration in a tropical bat species. In contrast to the previous case study which was a direct illustration of the MGF guidelines, this one shows the synergy that can be gained by combining several threads of research into the MGF framework.

#### Hibernation and rabies virus infection in big brown bats

The first part of our case study focused on rabies virus persistence in a big brown bat (*Eptesicus fuscus*) population roosting in buildings in Fort Collins, Colorado. In this study, modellers relied on a *post hoc* synthesis of field and laboratory data to develop a population-level model for the seasonal dynamics of rabies virus, which was then validated with independent data (George *et al*. 2011). Thus, this project effectively implemented Steps 3, 4 and 5 of the MGF framework (Fig. 3). The project was initiated by field and laboratory scientists aiming to estimate host demographic parameters that were previously unavailable. From the onset of the 5-year field phase of the project, empirical estimates of bat demographic and infection-related parameters were obtained (Step 3). Field work included radio-tracking of bats and permanently tagging (Wimsatt *et al*. 2005) several thousand individual bats at multiple colonies, which provided estimates of survival rates (O'Shea *et al*. 2011b). Reproductive rates were quantified by assessing the breeding status of captured females (O'Shea *et al*. 2010). The project also considered epidemiological parameters of the bat population, including determination of rabies seroprevalence and assessment of infection prevalence. Interpretation of serology data was helped by exposure experiments on captive big brown bats (Shankar *et al*. 2004; Davis *et al*. 2007; Turmelle *et al*. 2010) and a statistical model for estimating the rate of exposure based on seroprevalence and seroconversion data from marked bats (George *et al*. 2011).

The mathematical model consisted of three submodels that described the hibernation period (when no transmission occurs), pre-transmission period in early spring and the main transmission period (when transmission was assumed to follow a classical SEIR framework). Other structures representing alternative hypotheses were considered for the pre-transmission period where less was known. Combined results of demographic and serological sampling in the field, results of experimental exposure studies and information from the literature allowed modellers to estimate or bound model parameters. The model was validated with independent data from the study population, including population size (O'Shea *et al*. 2011a), size of the infectious class (George *et al*. 2011) and the timing of the peak number of rabies cases in different age classes (O'Shea *et al*. 2011a).

The model suggests that rabies virus is maintained in the population because the system is essentially in stasis during the hibernation period, which allows persistence of infection until the spring birth pulse (George *et al*. 2011). Although this project did not begin with the design of a mathematical model, the field and experimental biologists had an unusually clear conceptual model initially and worked closely with modellers as data collection was ending. Although, in hindsight, this project could have benefitted from an earlier involvement of the modellers, it has demonstrated how empirical researchers and modellers effectively iterated several steps of MGF *post hoc* (Fig. 3). The resultant model would not have been possible or compelling without the field and laboratory data generated at different stages of the project; the modelling allowed integration of empirical information to ensure that new insights were made with regards to rabies maintenance in the host population and tested alternative hypotheses regarding the pre-transmission season. In addition, this provided us with a starting point to develop a model for our next study.

#### Migration and LBV in straw-coloured fruit bats

The second part of our case study on bat-lyssavirus systems concerns Lagos bat virus (LBV), a lyssavirus that has been isolated from the African frugivorous bat *Eidolon helvum* (Boulger & Porterfield 1958). This project exemplifies how emerging infectious disease research can be undertaken when little prior knowledge exists. A high prevalence of antibodies against LBV had been detected in this migratory species (Hayman *et al*. 2008; Kuzmin *et al*. 2008), which led us to formulate three overarching questions. Given that most lyssaviruses were thought to have high lethality rates in any mammalian host they infect, could LBV remain endemic in *E. helvum*? How would the spatiotemporal structure of bat populations at the continental scale affect viral persistence? Could *E. helvum* act as a reservoir for zoonotic spillover into human and livestock populations? *Eidolon helvum* often roosts in enormous colonies reaching several million bats (Sørensen & Halberg 2001), which have been documented to form and disperse seasonally across sub-Saharan Africa, in both urban and rural environments. Based on this prior knowledge, we set out to address our three questions, with an initial focus on the capital of Ghana, Accra, home to a large colony of *E. helvum* in close contact with city-dwellers. We have concentrated our attention on two main factors: heterogeneity in host populations (e.g. variations in prevalence with location or age) and potential seasonal forcing of transmission caused by the seasonal migratory and reproductive behaviours of *E. helvum*. We have been addressing these issues following the MGF framework (Fig. 4) and using to our advantage the knowledge acquired through the big brown bat study.

Using the limited knowledge available on the life cycle of *E. helvum* (Mutere 1967) and the dynamics of other bat lyssaviruses (Mondul *et al*. 2003; Harris *et al*. 2006), we initiated the project with a simple ecological model (Fig. 4, Step 1), based on the hypotheses that LBV infection in *E. helvum* can be transmitted horizontally within bat populations and results in protective immunity, given the high seroprevalence detected. Given the lack of empirical information on those two hypotheses, the model-design process generated a large number of more specific questions concerning, in particular, the existence of protective maternal antibodies, the duration of immunity and the lethality of infection. The absence of quantitative information on the ecology and demography of the bats meant that demographic studies were also necessary. Thus, over a few years, we iteratively accrued data and parameter estimates through diverse field studies of the host, the pathogen and their environment, combined with a series of demographic and epidemiological mathematical models (Fig. 4, repeated loops between Steps 2 and 3).

We have been conducting in parallel field-based studies of wild populations, surveys of captive bats maintained in semi-natural conditions and laboratory-based development of immunological and virological assays (Hayman *et al*. 2011), all guided by and feeding back into the modelling framework. The large colony sizes of *E. helvum* roosts largely exceed the number of bats that can be studied using a traditional capture-recapture marking survey. Simulations based on expected survival rates suggested that radio-telemetry as a method of redetection of around 100 tagged bats in the colony over a whole season would give reliable estimates of survival (Hayman *et al*. 2012b). Other approaches, such as the estimation of ages using tooth-cementum ring analyses, have enabled us to estimate age-specific seroprevalence, and hence infection rates (Hayman *et al*. 2012a). To address the unresolved issue of protective acquired immunity in bats, a captive, wild-caught colony is now being studied. By sampling these bats regularly, we are seeking to determine whether serological parameters vary over time, whether all individuals are born susceptible and whether seropositive bats may be persistently infected. Alongside this ongoing empirical work, we are now adapting the seasonal model developed by George *et al*. (2011) for rabies virus in big brown bats, to LBV in *E. helvum*. Once data collection from captive bats is complete, we will be ready to move to Step 4 and fit our updated transmission model to empirical data.

## Discussion

We have presented a detailed framework for MGF and described, through case studies, different methods of implementation. Key differences between MGF and more commonly applied approaches to modelling ecological dynamics are the early-stage input from both modellers and biologists into study design, incorporation of multiple hypotheses and uncertainty about structure in the determination of the data required and the iterative approach between models and measurement. *Post hoc* modelling studies have an important role to play to complement traditional data analysis and generate new predictions. However, such studies tend to be limited in their scope and power if the modellers were not involved in study design. A lack of mutual understanding or communication between the modellers and the scientists who collected the data can cause lengthy adjustments of the model, can result in parts of the data being unusable due to their inadequate collection or reporting and may bring into question the reliability of the predictions generated. When undertaken properly, MGF allows the assessment of multiple system-specific hypotheses that relate to unobserved mechanisms, by combining information from different organisational levels (individual hosts, populations and landscapes). This information often comes from diverse sources, such as field surveys, laboratory experiments and surveillance, that can be integrated into the modelling framework. While we put fieldwork at the core of MGF as the fundamental source of data and observations on wildlife ecology, our framework can incorporate complementary data sources, such as experiments involving captive animals. As a result of this broader integration, the data gathered will be exploited to their full potential and will thus lead to richer and more reliable conclusions and predictions.

Beyond the technical aspects that we have described in detail, MGF provides a framework for adaptively managing the human dimension of interdisciplinary research collaborations. Indeed, the iterative process allows input from all disciplines at multiple points, helping to resolve or avoid altogether counter-productive situations where modellers are asked to develop a model without sufficient data or where field biologists are handed a model with inappropriate assumptions. However, even within this framework, there are critical capabilities that must be developed within the research group as a whole to ensure an efficient and durable collaboration. First, the group must define common goals and questions (Gorman *et al*. 2012), which is not as easy to achieve as it may sound. Too often in research, interdisciplinary collaborators draw targets around their current personal research interests rather than defining superordinate goals and then agreeing on the means to achieve them. The MGF approach essentially forces participants to develop shared questions and goals before the research is undertaken. Second, members of the collaboration need to develop ‘moral imagination’ which involves seeing the problems from the perspective of other stakeholders (Gorman *et al*. 2012). Often researchers from different fields will have fundamentally different perspectives on how to tackle a problem, aptly described by Gorman *et al*. (2001) who stated that ‘most scholars like to share frameworks about as much as they like to share toothbrushes’. Hence, the third capability, developing ‘trading zones’ (Galison 1997), is necessary for exchanging ideas or sharing data and resources. All three of these capabilities can be facilitated by ‘interactional experts’—individuals who understand enough of the disciplinary cultures and languages to facilitate a common language, common goals, shared mental models, exchange of knowledge and a shared framework for investigation (Gorman 2010). Such expertise has traditionally been gained over long periods of personal collaborations, but more opportunities are now available for early-career training through formal courses and workshops. For example, in MGF, interactional experts would include veterinarians with post-graduate training in mathematical modelling, immunologists with training in ecology or mathematicians with a training in epidemiology. Both offer and demand for such interdisciplinary training need to be encouraged.

Now is an exciting time to implement MGF approaches. There is increasing emphasis on the need for multi- or interdisciplinary studies of many systems, particularly regarding the emergence of infectious disease threats to biodiversity and public health (Wolfe *et al*. 2007; Jones *et al*. 2008). Many national and international funding bodies have started to support actively the integration of empirical research with modelling, or model-guided predictions, not only in their core programmes but also increasingly in direct response to emerging epizootics (e.g. white nose syndrome in North American bats) or zoonoses (e.g. pandemic swine influenza). One area where real progress can be made at low additional cost through MGF approaches is pathogen or disease surveillance—a point apparently overlooked by Kuiken *et al*. (2005) in their call to arms to tackle emerging zoonotic infections. Such approaches to surveillance would potentially increase the return on investment by addressing traditional surveillance questions as well as more mechanistic ones.

Although we have focused our attention on wildlife infections, the MGF framework would be equally useful in other areas of applied ecological research where mechanistic models could help devise quantitative predictions for intervention, as indicated by retrospective reviews, e.g. on pest management (Murdoch *et al*. 2006). Potential applications include the management of invasive species, conservation of biodiversity in the face of climate change or the sustainability of human exploitation of natural resources. Advice to policy makers in these fields all too often relies on ‘expert opinion’, a euphemism for the subjective synthesis of vast amounts of scientific evidence and personal experience. By providing a rigorous framework for the construction of such syntheses, the MGF approach has the potential to become a standard for evidence-based environmental policies. Explicit embedding of MGF approaches into policy directed programmes can be achieved using frameworks such as participatory impact pathways analysis (Alvarez *et al*. 2010). A clearly defined pathway towards a successful outcome can provide incentives for all scientists to engage in what for many could be an unfamiliar and uncomfortable, albeit hugely exciting, process.
